# The effect of xylitol chewing gums and candies on caries occurrence in children: a systematic review with special reference to caries level at study baseline

**DOI:** 10.1007/s40368-024-00875-w

**Published:** 2024-03-02

**Authors:** K. Pienihäkkinen, A. Hietala-Lenkkeri, I. Arpalahti, E. Söderling

**Affiliations:** Institute of Dentistry, University of Turku, Lemminkäisenkatu 2, 20520 Turku, Finland

**Keywords:** Dental caries, Xylitol, Polyol, Chewing gum, Caries prevention

## Abstract

**Purpose:**

A systematic review of published data was carried out to assess the caries-preventive effects of xylitol chewing gums and candies in children.

**Methods:**

Electronic and hand searches were performed to find clinical studies on the effects of xylitol chewing gums and candies on dental caries in children (≤ 18 years). Prospective randomised or controlled clinical trials published before 2023 were included in the review.

**Results:**

The initial search identified 365 titles to be evaluated. After applying inclusion and exclusion criteria, 15 articles with either fair or low quality were reviewed. Nine articles studied chewing gums, five candies, and one both of them. In the ten evaluated xylitol chewing gum studies xylitol consumption significantly reduced caries occurrence when compared with no treatment or a placebo polyol gum. The effect was clinically significant in studies with high or moderate caries level at study baseline. The results also suggested that the caries-reducing effect of xylitol gums may differ from sorbitol/polyol gums. In five of the six heterogenous xylitol candy studies, no caries-reducing effect was found independent of caries level. In addition to caries level, also the daily xylitol dose was a confounding factor.

**Conclusion:**

The present findings suggest that the caries-reducing effect of adding xylitol chewing gum to the daily diet has been well demonstrated in children and adolescents with high or moderate caries level at study baseline. Xylitol gum use could benefit subjects with active incipient caries lesions on smooth tooth surfaces.

## Introduction

Xylitol is a naturally occurring five-carbon polyol sweetener which promotes mineralization by increasing the flow of saliva, especially in association with the use of chewing gum. This effect is shared by other polyol sweeteners such as sorbitol and maltitol. However, xylitol differs from them in that it is not fermented by the oral microbiota (Havenaar et al. 1978). Xylitol consumption has also been shown to reduce the acid production potential of plaque (Campus et al. 2009; Splieth et al. 2009). Several randomised clinical trials suggest that habitual xylitol chewing gum consumption is likely to decrease counts of caries-associated mutans streptococci and that the reduction differs from that found for sorbitol/polyol gum (Milgrom et al. 2006; Campus et al. 2009; Söderling and Pienihäkkinen 2020). Habitual xylitol gum chewing appears also to reduce the amount of dental plaque (Nasseripour et al. 2022, Söderling and Pienihäkkinen 2022). These specific “xylitol-effects” have been attributed, amongst others, to growth inhibition of mutans streptococci and less adhesive plaque due to reduced amounts extracellular polysaccharides in the plaque (Söderling 2009). Also, the mother–child studies, demonstrating a decrease in the early transmission of mutans streptococci and caries occurrence in the children following maternal xylitol consumption, suggest specific effects for xylitol (Li and Tanner 2015). Clinical xylitol caries trials have been carried out since the 1970s, when the Turku Sugar Studies demonstrated that total substitution of sucrose with xylitol resulted in practically no new caries lesions (Scheinin et al. 1975).

Earlier systematic reviews on the effects of xylitol or sugar-free chewing gums on dental caries have resulted in a great variation of conclusions: three systematic reviews suggested that the evidence for the use of sorbitol or xylitol in chewing gum is inconclusive (Lingström et al. 2003) or insufficient to determine whether various xylitol products had any caries-preventive effect (Riley et al. 2015) or too weak to support use of xylitol chewing gum (Mota et al. 2021). Three reviews supported or recommended using polyol-containing chewing gum (Deshpande and Jadad 2008), or sucrose-free chewing gum or xylitol lozenges (Rethman et al. 2011) for caries prevention, or xylitol wipes as an adjunct for caries control in young children (Wang et al. 2017). An additional six reviews reported at least some caries-reducing effect for sugar-free chewing gums (Mickenautsch et al. 2007; Newton et al. 2020) and for xylitol products (Antonio et al. 2011, Janakiram et al. 2017, Marghalani et al. 2017, ALHumaid and Bamashmous 2022). In these previous systematic reviews, the focus has been on the evaluation of the clinical effects of xylitol or other polyols, or on dietary factors in general. The research questions have varied, and in several reviews, the inclusion criteria have been quite strict. This has resulted in a very low number of studies to be included and in great variation between the studies accepted for the analysis. On the other hand, many of these reviews include critical remarks on the heterogeneity of the studies, regarding the age of subjects as well as the mode and duration of polyol delivery.

With the exception of Antonio et al. (2011), no other review has considered the significance of the caries level at study baseline and its role in the outcomes of the evaluated studies. It is noteworthy that the level of caries experience has changed considerably since the 1970s. In very severe caries conditions, practically all children and adolescents had dental decay and nearly all teeth were affected by caries. With decreasing severity of the disease there are also changes in the predilection sites of the decay. Thus, first the incisors and next the premolars, are no longer affected (Massler et al. 1954; Ruiken et al. 1986). On a tooth surface level, caries on smooth surfaces decreases first and the most, whereas the caries on the occlusal surfaces of first molars seems to be reduced the least. As a result, in low-caries conditions, tooth decay in children and adolescents is largely a phenomenon of occlusal pits and fissures of the posterior teeth (Selwitz et al. 2007; Nørrisgaard et al. 2016).

Our aim was to describe and evaluate the literature published during 1974–2022 in relation to the caries-preventive effect of xylitol chewing gums and candies in children. We did not limit the review to randomised trials only, since it is evident that trials conducted in the 1970s, 1980s and even 1990s will have no proper randomisation, or at least there would be problems with it. We did not even plan to do a meta-analysis, since it was to be expected that the studies within this broad time frame will be very heterogenous. Instead, the enormous change in the caries levels of study populations would allow a wider perspective in the description and understanding the studies’ outcomes. With this systematic review we aimed to answer the defined research questions: (1) can the consumption of xylitol chewing gum and/or candies reduce occurrence of dental caries in children, (2) what is the importance of the caries level of the children for the outcome, and (3) are the effects specific to xylitol?

## Materials and methods

The Preferred Reporting Items for Systematic Reviews and Meta-Analyses (Page et al. 2021:PRISMA STATEMENT) was used as a guideline in the present systematic review. The review was registered in PROSPERO (CRD42022376771) before starting the data collection.

### Information sources and research strategies

The research question for the present systematic review was formulated using PICO characteristics (Patients, Intervention, Control, Outcome), as follows: in children (0–18 years) (P), are xylitol chewing gums and/or candies (I), compared with a control (no gum/candy, a placebo gum/candy) (C), effective in decreasing caries occurrence (during the follow-up the reduction in the caries increment or in the proportion of subjects/teeth/tooth surfaces with new dental decay) (O)?

The search to identify all the relevant, published studies was conducted using three databases: PubMed, Embase and the Cochrane Library. Grey literature was searched on ClinicalTrials.gov. A hand search was conducted in the reference lists of previous systematic reviews close to the present topic. The searches were conducted in December, 2022 and checked for additional literature on January 3–5, 2023. The search thus covered the years 1974–2022.

The following terms were used in the search for xylitol studies:

(xylitol* OR “xylitol” [Mesh]) AND (caries* OR “dental caries” [Mesh]) NOT (“Adult” [Mesh] OR “Aged” [Mesh] OR adult*) AND (“Randomised Controlled Trial” [Publication Type] OR “Controlled Clinical Trial” [Publication Type] OR “field study*” OR randomised*[tw] OR randomised*[tw] OR placebo[tw] OR randomly*[tw] OR trial[tw] OR groups[tw]) NOT (animals[Mesh] NOT humans[Mesh])– PubMed.

(xylitol* OR ‘xylitol’/exp) AND (caries* OR ‘dental caries’/exp) NOT (‘adult’/exp OR ‘aged’/exp OR adult*) AND (‘controlled clinical trial’/exp OR ‘controlled study’/exp OR “field study*” OR randomised* OR randomised* OR placebo OR randomly* OR trial OR groups) NOT (‘animal’/exp NOT ‘human’/exp)– Embase.

xylitol* AND caries* NOT (adult*) AND (“field study*” OR randomised* OR randomised* OR placebo OR randomly* OR trial* OR groups) NOT animal*– Cochrane.

### Study inclusion and exclusion criteria

Prospective randomised controlled clinical trials (RCT) and controlled clinical trials (CCT) conducted in children (≤ 18 years) were included in the review. For the evaluation the study had to be available in English. The aim of the included trials was to study the effect of xylitol chewing gum and/or candies on caries occurrence. Caries was either the primary or secondary outcome measure in the evaluated studies. The comparison/control was no gum/candy or a placebo gum/candy. The daily dose of the polyol had to be available to meet the inclusion criteria. Xylitol had to be the polyol, with a concentration of 50% or more of total polyols in the product. The baseline caries level had to be available. Minimum duration of the study had to be: 1 year in < 7-year-old children; 2 years in 7–18-year-olds.

Exclusion criteria used when evaluating titles and abstracts: dental caries was not an outcome of the study; studies in subjects > 18 years; no proper control; in vitro studies, reviews, comments or study protocols; the polyol vehicles were oral sprays or rinses, toothpastes, pacifiers, milk, wipes or varnishes; the xylitol products contained components like fluoride, carbamide or polydextrose; mother-to-child mutans streptococci transmission studies; the study was not available in English.

Exclusion criteria used when evaluating full-text articles: in one study the age range of the subjects was 10–27 years (Honkala et al. 2006).

### Study selection and data extraction

Screening of the records was performed after duplicate removal independently by the reviewers (KP, ES, AH, IA). Two of them (ES, KP) have been calibrated during the evaluation and analysis process of similar systematic reviews between the years 2020 and 2022. The other two (AH, IA) had been calibrated earlier with KP during their PhD studies. The review team members scanned the titles and, when needed, read the abstract. The team members independently decided which studies in their opinion fulfilled the criteria for full-text review.

Two of the reviewers (ES, KP) collected the data from the articles chosen for the full-text review. The following data were collected: author and year of publication, study site, number and age of participants, study design, intervention and controls, outcome measure, caries at baseline, caries increment, main results. In case, the design in the evaluated study included several xylitol subgroups, the subgroup with the longest duration was selected for the present analysis. In studies performed with 100% polyol products vs. polyol mixture products, the 100% products with the highest recommended consumption frequency were chosen for evaluation. For baseline caries and caries increment values, preferably indices measuring dentinal caries or localised enamel breakdown (corresponding to ICDAS 3–6 decay) were selected. The baseline caries was classified into three categories: high (H), moderate (M) and low (L), based primarily on reported dmfs/DMFS group mean or, when not available, on the proportion of subjects with caries experience. The categorization was in accordance with Petersen et al. (2003). As the measure of preventive effect, two of the reviewers (KP, AH) calculated the prevented fraction (PF) based on reported results. PF was the proportion of caries reduction (= mean caries increment in control group – mean caries increment in intervention group) of mean caries increment in control group and expressed in percentage. Any disagreements were resolved by discussion amongst all reviewers.

### Assessment of methodological quality and risk-of-bias

Even though not all the selected trials were randomised, we used the Cochrane risk-of-bias tool for randomised trials (RoB 2) in the evaluation of the selected trials (Higgins et al. 2022). The reviewers independently evaluated the included full-length articles and, based on mutual agreement, eliminated discrepancies between each individual assessment.

The studies were appraised according to the following aspects: selection, performance, detection, attrition, reporting, and funding bias. Each aspect was classified as having either low, high or unclear risk-of-bias. The bias was estimated to be unclear, for example, if the study was randomised but details on randomisation were not given. Also, when information not found in the paper was found, e.g. in the theses of the authors, the bias was classified as unclear. The overall level of risk for each study was classified as low (all quality items were met: high quality), unclear (unclear risk-of-bias for one or more domain: fair quality), or high (high risk-of-bias for one or more domain: low quality) (Wang et al. 2017; Higgins et al. 2022).

The theses of Isokangas (1987), Kovari (2002) and Hietala-Lenkkeri (2016) were searched for data missing from the publications included in this evaluation (Isokangas et al. 1988; Kovari et al. 2003; Lenkkeri et al. 2012). Number 6, Volume 43 (1985) of Acta Odontol Scand was used to find more information on the Scheinin et al. (1985) trial. The 1-year results by Kandelman and Gagnon (1987) were used when evaluating the 2-year findings in this review (Kandelman and Gagnon 1990).

## Results

### Study selection

**Fig. 1:**
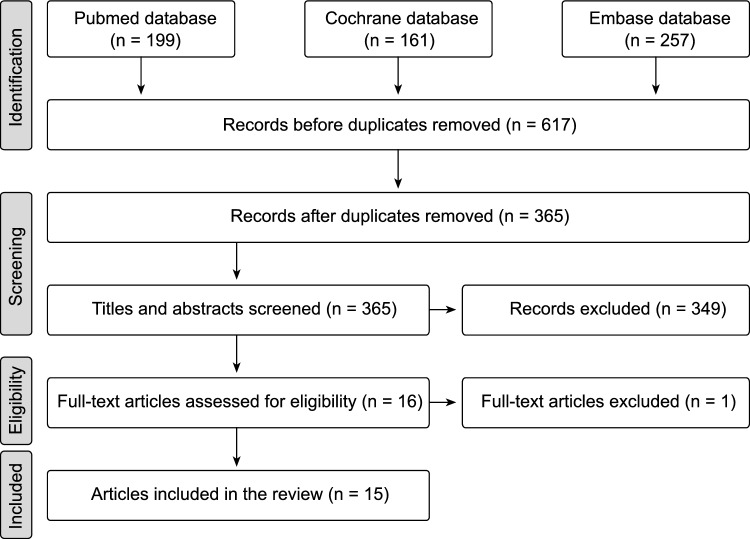
Flow chart

In the search for xylitol articles, a total of 617 titles were screened for relevance (199 PubMed, 257 Embase, 161 Cochrane). Exclusion of duplicates left 365 titles to be evaluated. Based on the information in the abstracts, 349 articles were removed. When full-text articles were assessed for eligibility, one article was excluded, which resulted in 15 articles to be reviewed (Fig. 1).

### Study characteristics

All trials included in the review were either controlled clinical trials or randomised controlled trials published between 1985 and 2015 (Scheinin et al. 1985; Isokangas et al. 1988; Kandelman et al. 1988; Kandelman and Gagnon 1990; Mäkinen et al. 1995; Mäkinen et al. 1996; Alanen et al. 2000; Machiulskiene et al. 2001; Kovari et al. 2003; Oscarson et al. 2006; Stecksén-Blicks et al. 2008; Lenkkeri et al. 2012; Campus et al. 2013; Honkala et al. 2014; Lee et al. 2015). All studies reported the age of the children (age range from 2 to 12 years), sample size (ranging from 118 to 744), and study duration (from 2 to 6 years). The delivery modalities included chewing gums and/or confectionery, candies, lozenges and gummy bears. The two studies (Scheinin et al. 1985; Kandelman et al. 1988) in which confectionery was used as the delivery method for xylitol, the main component of the confectionery was chewing gum. Thus, these studies were evaluated as chewing gum studies. Lozenge and gummy bear studies were evaluated as candy studies. The number of subjects refers to those subjects who completed the study within the groups of the present comparisons (Table 1). In the majority of the studies, the primary outcome measure was caries (Table 1). In two studies the caries indices included also enamel caries (Kandelman et al. 1988; Kandelman and Gagnon 1990). In one study, the primary outcome measure was the colonization of mutans streptococci (Oscarson et al. 2006).

**Table 1:** Summary of the included studies

	Subjects, *n*	Study design	Intervention	Control	Outcome measure	BL caries	Caries increment	Prevented fraction	Results
**Permanent teeth**									
Scheinin et al. 1985; Budapest, Hungary	7–10-yr-old children, *n* = 423 (institutionalised)	Controlled clinical trial, 3 yrs; caries pom	X confectionery, 14–20 g/d	No confectionery (C), F in milk or water (F)	DMFS	X/F/C: 3.2/2.2/2.1 (H)	X/F/C: 2.3/3.4/3.5	X/F: 32%; X/C: 34%	The caries increment was lower in the X group compared to the F and C groups (*p* < 0.001)
Isokangas et al. 1988; Ylivieska, Finland	11–12-yr-old children, *n* = 324	Controlled clinical trial, 2 yrs; caries pom	X gum (65%), 10 g/d, 3xd	No gum (C)	DMFS	X/C: 4.1/4.8 (M)	X/C: 1.1/2.0	45%	The caries increment in the X group was significantly lower than that in the C group (*p* < 0.001)
Kandelman et al. 1988; French Polynesia	6–12-yr-old children, *n* = 468	Controlled clinical trial, 2 yrs 8 mo; caries pom	X confectionery, 20 g/d	No confectionery (C)	DMFS including enamel caries	X*/C: 7.2/5.6 (H)	X*/C: 4.5/7.2	37%	Partial substitution of dietary sugar with X confectionery decreased caries increment significantly (*p* < 0.001)
Kandelman & Gagnon 1990; Montreal, Canada	8–9-yr-old children, *n* = 184	Controlled clinical trial, 2 yrs; caries pom	X gum (65%), 3.4 g/d, 3xd	No gum (C)	DMFS including enamel caries	X/C: 6.3/5.7 (H)	X/C: 2.1/6.1	66%	The progression of caries was significantly lower in the X gum group compared to the no-gum group (*p* < 0.01)
Mäkinen et al. 1995; Belize City, Belize	10-yr-old children, *n* = 400	Controlled, double-blind trial, 3 yrs 4 mo; caries pom	X pellet gum (Xp) (65%), stick gum (Xs) (60%), 9 g/d, 5xd	S pellet gum (Sp) (65%), 9g/d, 5xd, no gum (C)	DMFS	Xp/Xs/Sp/C: 5.1/6.1/5.4/4.3 (H)	Xp/Xs/Sp/C: -0.7/0.5/3.7/5.0	Xp/C: 114%, Xs/C: 90%, Xp/Sp: 26%	The X pellet and stick gums showed significantly lower caries increments compared to the S pellet or no-gum groups (*p* < 0.001)
Alanen et al. 2000; Estonia	10-yr-old children, *n* = 334	Cluster-randomised, controlled, blinded study, 3 yrs; caries pom	X gum (65%), X lozenges (X 49%, MAL 47.5%), 5 g/d, 3xd	No product (C)	DMFS	X gum/X candy/C: 1.8/1.6/2.2 (M)	X gum/X candy/ C: 1.9/1.7/4.4	X gum/C: 58%, X candy/C: 61%	The caries increments did not differ between the two X groups, but were significantly lower than that of the C group (*p* < 0.01)
Machiulskiene et al. 2001; Kaunas, Lithuania	9–14-yr-old children, *n* = 179	School-based randomised, controlled, double-blind study, 3 yrs; caries pom	X gum (62%), 3 g/d, 5xd	S gum, placebo gum (PG), no gum (C)	DMFS	X/S/PG/C: 13.2/14.1/ 15.3/14.3 (all caries stages (H)	X/S/PG/C: 3.4/4/4.3/5.3	X/C: 36%, S/C: 13%, PG/C: 19%	The 3-yr caries increment (cavitated stages) was lower in X than C group (*p* < 0.05). S and PG did not differ from C
Stecksén-Blicks et al. 2008; Umeå, Sweden	10–12-yr-old children, *n* = 120 (high-caries risk)	Controlled, blinded study, 2 yrs; proximal caries pom	X lozenges, 2.5 g/d, 3xd	No lozenge, OHE/F-varnish 2xyr (C)	DMFS proximal, radiographic examination	X/C: 0.5/0.7 (M)	X/C: 0.7/0.7	0%	The X lozenge consumption did not prevent approximal caries
Lenkkeri et al. 2012; Kotka, Finland	10-yr-old children, *n* = 200	Cluster-randomised, controlled, double-blind trial, intervention 2 yrs, follow-up 4 yrs; caries pom	X lozenge (X 49%, MAL 47.5%), 4.7 g/d, 3xd	No lozenge (C)	DMFS	X/C: 0.4/0.3 (L)	X/C: 1.6/1.5	-7%	The X lozenge use did not add to the effect of comprehensive caries prevention
Campus et al. 2013; Sassary, Italy	7–9-yr-old children, *n* = 148 (high-caries risk)	Randomised, controlled, double-blind trial, intervention 6 mo, follow-up 2 yrs; caries pom	X gum (X 37%, S 18%, MAL 10%, MAN 7%), 11.6 g/d, 5xd	Isomalt gum (IG) (isomalt 30%, S 18%, MAL 16%, MAN 7%), 5xd	Proximal DFS for permanent first molars	X/C: 57%/56% (H)	Additional % of children with decayed first molars: X/IG 1.4%/10.3%	86%	The increase in the proportion of children with decayed permanent first molars was lower in the X than the IG group (*p* < 0.01)
**Permanent and primary teeth**									
Kovari et al. 2003; Savonlinna, Finland	3–6-yr-old children, *n* = 744 (kindergarten)	Randomised, controlled study, intervention 8–30 mo, final examination at 9 yrs; caries pom	X gum (65%), 2.5 g/d, 3xd	Toothbrushing 1xd after lunch (C)	dmf/DMF > 0	X/C: 8%/6% (L)	Additional % of children having dmf/DMF > 0: X/C 35%/45%	22%	A statistically significant, but clinically small difference was found in favour of the X group. NNT = 12.2 (6.5–108.7)
Honkala et al. 2014; Tartu, Estonia	7–9-yr-old children, *n* = 252	Randomised, controlled, double-blind trial, 3 yrs; caries pom	X (90%) candy, 7.5 g/d, 3xd	S (90%) candy, 7.5 g/d, 3xd	d4-6mfs + D4-6MFS	X/S: 11.2/12.7 (M/H)	% of surfaces developing dentin caries: X/S 2.0%/1.7%	-18%	No significant difference in dentin caries development between the X and S groups
Lee et al. 2015; Cleveland, USA	5–6-yr-old children, *n* = 260 (high-caries risk)	Cluster-randomised, controlled, double-blind study, intervention 9 mo, follow-up 2 yrs 6 mo; caries pom	X gummy bears, 7.8 g/d, 3xd	Inulin gummy (C) bears, 3xd	d3-6mfs, D3-6MFS	X/C: d3-6mfs: 6.3/4.3, D3-6MFS: 0.0/0.0 (H)	X/C: d3-6mfs: 5.0/4.0, D3-6MFS: 0.4/0.5	dmfs: -25%, DMFS: 21%	The caries increment in primary and permanent teeth in both groups was low, no difference between the groups
**Primary teeth**									
Mäkinen et al. 1996; Stann Creek, Belize	6-yr-old children, *n* = 335	Controlled, double-blind cohort study, 2 yrs; caries pom	X pellet gum (Xp) (60.5%) and X stick gum (Xs) (65%), 10 g/d, 5xd	S pellet (Sp) (65%) and S stick (Ss) (61%) gum, 10g/d, 5xd, no gum (C)	dmfs	Xp/Xs/Sp/Ss/C: 12.2/11.0/ 10.5/12.8/ 12.8 (H)	Xp/Sp/C: 1.8/2.4/4.9, Xs/Ss/C: 2.6/3.7/4.9	Xp/C: 63%, Xs/C: 47%, Xp/Sp:25%, Xs/Ss: 30%	All chewing gums reduced caries compared to no gum (Xp, Xs, Sp *p* < 0.001; Ss *p* < 0.05)
Oscarson et al. 2006; Lycksele, Sweden	2-yr-old children, *n* = 118	Randomised, controlled, blinded study, intervention 18 mo, follow-up 2 yrs; caries som	X lozenge (X 49%, MAL 47.5%), 0.5-1g/d, 1-2xd	No lozenge (C)	dmfs	X/C: 7%/6% (L)	X/C: 0.8/1.2	33%	Caries increment in both groups low, no significant differences between groups

*F* fluoride, *C* control, *X* xylitol, *S* sorbitol, *MAL* maltitol, *MAN* mannitol, *yr* year, *yrs* years, *mo* months, *d* days, *pom* primary outcome measure, *som* secondary outcome measure, *OHE* oral health education, *DMFS* dmfs, *DFS* the number of decayed (D/d), missed (M/m) and filled (F/f) surfaces for permanent/deciduous teeth, *BL* baseline caries level, high (H), moderate (M), low (L), *NNT* number needed to treat (confidence interval), *PG* gum sweetened with artificial sweeteners

*Geometric mean of caries indices of two xylitol subgroups

In the trial by Scheinin et al. (1985), the children were institutionalised, predominantly orphans, but also children with hearing or vision impairment in two of eleven institutions. In three studies, children with systemic diseases or antibiotic use (Campus et al. 2013), or systemic diseases (Lenkkeri et al. 2012), or disabled children and individuals with complicated chronic diseases or intellectual disability (Stecksén-Blicks et al. 2008) were excluded from the study. In the study by Lee et al. (2015), the children included in the study were reported to be healthy, free of stomach illnesses and without strict dietary restrictions. In the rest of the trials, no health issues were reported (Isokangas et al. 1988; Kandelman et al. 1988; Kandelman and Gagnon 1990; Mäkinen et al. 1995; Mäkinen et al. 1996; Alanen et al. 2000; Machiulskiene et al. 2001; Kovari et al. 2003; Honkala et al. 2014; Lee et al. 2015) or the children were reported to be healthy (Oscarson et al. 2006).

**Fig. 2:**
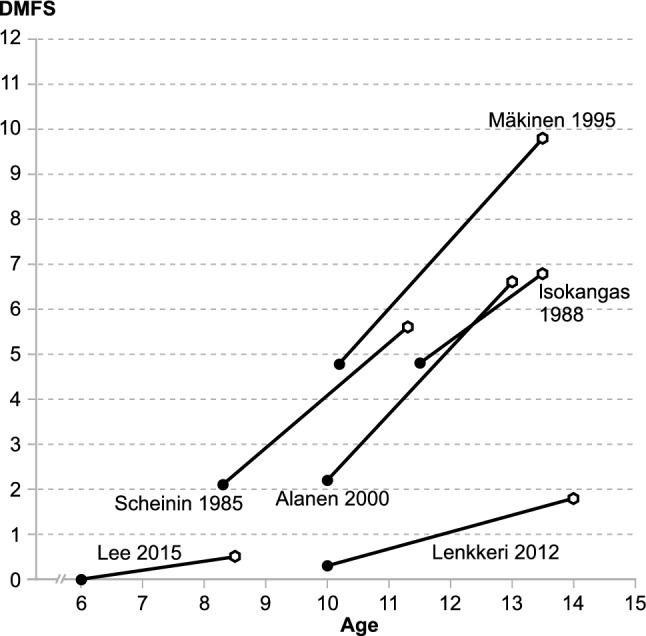
The mean baseline DMFS and incremental DMFS values in the control group in six evaluated studies in relation to average initial age of the subjects and the follow-up period. For these six studies DMFS index (cavitated level) was available

The trials were conducted in children with a high or moderate level of caries, except for three studies in which the children’s caries level was low (Kovari et al. 2003; Oscarson et al. 2006; Lenkkeri et al. 2012). In three studies, high-caries risk subjects had been screened using individual indicators of high-caries risk (Stecksén-Blicks et al. 2008; Campus et al. 2013) or by including children of low-income district, minority parents/caregivers with a high-caries occurrence (Lee et al. 2015). In the trial by Lee et al. (2015), the subjects were young. Thus the number of permanent teeth/tooth surfaces at risk was very low. Except for the above three studies with screened subjects, the caries level of the children participating in the trials corresponded to the DMFT levels reported for the country in question (WHO CAPP). The DMFS index (cavitated level) of permanent teeth was available for six studies (Scheinin et al. 1985; Isokangas et al. 1988; Mäkinen et al. 1995; Alanen et al. 2000; Lenkkeri et al. 2012; Lee et al. 2015). The average baseline DMFS and incremental DMFS values within the control groups illustrates well the great variation between the evaluated studies (Fig. 2) in relation to baseline caries, the intensity of caries progression, the follow-up times, as well as the age of subjects.

Xylitol products were not replacing other sugar intake. In practise, all studies analysed involved xylitol use added to a normal diet, even though some authors used terms like “partial substitution” or “replacement of sucrose” in their text (Scheinin et al. 1985; Kandelman et al. 1988). Two studies registered consumption of sugar-containing products and found no differences between the xylitol and control groups (Oscarson et al. 2006; Campus et al. 2013). Even in the Mäkinen et al. (1995) trial in which the children consumed high amounts of sucrose on daily basis, no attempt to interfere with this consumption was made.

### Quality assessment of the selected studies

**Fig. 3:**
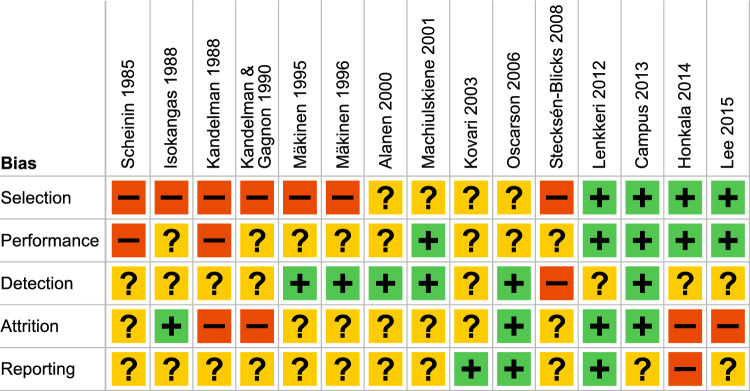
Risk of bias summary

Figure 3 summarises the risk-of-bias in the evaluated studies. The risk-of-bias assessment revealed that six studies had an unclear risk-of-bias (Alanen et al. 2000; Machiulskiene et al. 2001; Kovari et al. 2003; Oscarson et al. 2006; Lenkkeri et al. 2012; Campus et al. 2013), and the rest of the studies were scored as having a high risk-of-bias (Scheinin et al. 1985; Isokangas et al. 1988; Kandelman et al. 1988; Kandelman and Gagnon 1990; Mäkinen et al. 1995; Mäkinen et al. 1996; Stecksén-Blicks et al. 2008; Honkala et al. 2014; Lee et al. 2015).

Eight of the evaluated trials were randomised (Alanen et al. 2000; Machiulskiene et al. 2001; Kovari et al. 2003; Oscarson et al. 2006; Lenkkeri et al. 2012; Campus et al. 2013; Honkala et al. 2014; Lee et al. 2015), the rest were classified as controlled clinical trials (Scheinin et al. 1985; Isokangas et al. 1988; Kandelman et al. 1988; Kandelman and Gagnon 1990; Mäkinen et al. 1995; Mäkinen et al. 1996; Stecksén-Blicks et al. 2008). In sugar substitute caries trials, using, for example, classroom or school-based randomisation is a practical way to conduct such studies, and when confounding variables such as socioeconomic factors are taken into consideration, it can produce comparable groups at baseline (Lenkkeri et al. 2012). In some of the evaluated studies, however, the groups differed at baseline in relation to age (Scheinin et al. 1985; Mäkinen et al. 1995, 1996; Machiulskiene et al. 2001) or caries (Scheinin et al. 1985; Isokangas et al. 1988; Mäkinen et al. 1996; Lee et al. 2015), increasing the risk for selection bias. On the other hand, baseline differences were not tested in two studies (Kandelman et al. 1988; Alanen et al. 2000). In Stecksén-Blicks’ (2008) trial the randomised setup allowed comparison of the fluoride effect, but not the xylitol effect. Consequently, the study was classified as a CCT. In one trial (Oscarson et al. 2006) the subjects were randomised on an individual level, but details on the randomisation were not given. Apparently only in the double-blind trial by Campus et al. (2013), the participants were randomised on an individual level using computer-generated randomisation.

Caries was the primary outcome in all but one study (Oscarson et al. 2006). Thus, it was important that the caries registrations were performed blinded. The caries registrations were not blinded in three trials (Scheinin et al. 1985; Kandelman et al. 1988; Kandelman and Gagnon 1990), and only the final caries registrations were blinded in two trials (Isokangas et al. 1988; Kovari et al. 2003). Problems with allocation concealment and blinding are also inevitable when the control group does not chew gum or consume confectioneries (Scheinin et al. 1985; Isokangas et al. 1988; Kandelman et al. 1988; Kandelman and Gagnon 1990; Alanen et al. 2000; Kovari et al. 2003; Stecksén-Blicks et al. 2008; Lenkkeri et al. 2012; Oscarson et al. 2006).

Detection bias was low in six studies (Mäkinen et al. 1995, 1996; Alanen et al. 2000; Machiulskiene et al. 2001; Oscarson et al. 2006; Campus et al. 2013). In two studies (Stecksén-Blicks et al. 2008; Honkala et al. 2014), the outcome measure was not sensitive to detecting differences between the experimental groups. In the study of Stecksén-Blicks et al. (2008), the registration of caries was based on radiographs and only proximal caries was measured. A high number of surfaces which could not be assessed due to overlaps or other technical reasons were coded as healthy. In addition, the observed imbalance of caries indices at baseline was not considered during the statistical analyses creating a high risk-of-bias (Stecksén-Blicks et al. 2008). In the study of Honkala et al. (2014), the selected outcome measure (dmfs + DMFS) was not comparable with the measures of other studies. In seven studies the detection bias was unclear: the subjects were young and had a low number of permanent teeth resulting in a relatively low number of surfaces at risk, the examiners had not been calibrated or statistical analyses were not used to balance the differences in baseline values (Scheinin et al. 1985; Isokangas et al. 1988; Kandelman et al. 1988; Kandelman and Gagnon 1990; Kovari et al. 2003; Lenkkeri et al. 2012; Lee et al. 2015).

Attrition may be a problem in long-term caries trials. It was low in four studies, thus resulting in a low attrition bias (Isokangas et al. 1988; Oscarson et al. 2006; Lenkkeri et al. 2012; Campus et al. 2013). The attrition bias was unclear in seven studies (Scheinin et al. 1985; Mäkinen et al. 1995, 1996; Alanen et al. 2000; Machiulskiene et al. 2001; Kovari et al. 2003; Stecksén-Blicks et al. 2008). The attrition percentage was high and/or had not been taken into consideration in the statistical analyses in four trials resulting in high attrition bias (Kandelman et al. 1988; Kandelman and Gagnon 1990; Honkala et al. 2014; Lee et al. 2015).

The high attrition in long-term caries trials can be offset by including those subjects who completed the study in the analyses and doing a proper dropout analysis. Not only the older trials but also some newer ones, did not analyse the effect of the attrition, which led to an unclear risk of reporting bias (Kandelman et al. 1988; Kandelman and Gagnon 1990; Mäkinen et al. 1995; Stecksén-Blicks et al. 2008; Lee et al. 2015). Either no problems with reporting bias (Kovari et al. 2003; Oscarson et al. 2006; Lenkkeri et al. 2012) or only minor problems (Scheinin et al. 1985; Isokangas et al. 1988; Mäkinen et al. 1996; Alanen et al. 2000; Campus et al. 2013) were detected in eight studies. In the trial of Machiulskiene et al. (2001), the authors ignored that the 3-year caries increment for cavitated stages showed a significantly lower caries increment in the xylitol group compared to the no-gum control group (*p* < 0.05). This finding was not discussed by the authors, thus creating an unclear/moderate risk of reporting bias. The trial of Honkala et al. (2014) focussed on reporting the results on erythritol candies compared to xylitol and sorbitol candies. The study did not compare dropouts with those who completed the study in relation to baseline values. This fact, combined with the high attrition, leads to high risk of reporting bias.

Clinical trials with sugar substitutes are in practise difficult to conduct without some kind of industrial support. Conflict of interest was not registered in the older trials, however, information on funding was available for all studies. According to the information provided, two trials reported no conflicts of interest and were carried out without funding from xylitol or confectionary producers (Campus et al. 2013; Lee et al. 2015). In eight trials the projects had received the tested products as gifts from xylitol confectionery producers, creating an unclear risk-of-bias (Isokangas et al. 1988; Kandelman and Gagnon 1990; Alanen et al. 2000; Machiulskiene et al. 2001; Kovari et al. 2003; Oscarson et al. 2006; Stecksén-Blicks et al. 2008; Lenkkeri et al. 2012). Five trials received funding from xylitol or xylitol confectionery producers which was considered to create a moderate or high risk-of-bias (Scheinin et al. 1985; Kandelman et al. 1988; Mäkinen et al. 1995, 1996; Honkala et al. 2014).

### Study outcomes

Chewing gum was used as a delivery vehicle in ten of the 15 studies evaluated (Scheinin et al. 1985; Isokangas et al. 1988; Kandelman et al. 1988; Kandelman and Gagnon 1990; Mäkinen et al. 1995; Mäkinen et al. 1996; Alanen et al. 2000; Machiulskiene et al. 2001; Kovari et al. 2003; Campus et al. 2013). Two of these (Scheinin et al. 1985; Kandelman et al. 1988) had also other confectionery in the intervention, the maximum use of xylitol per day being relatively high. In six studies, lozenges (Oscarson et al. 2006; Stecksén-Blicks et al. 2008; Lenkkeri et al. 2012) or candies (Alanen et al. 2000; Honkala et al. 2014) or gummy bears (Lee et al. 2015) were used (Table 1).

All ten xylitol chewing gum studies, analysing a total of 3,466 subjects, reported a statistically significant preventive effect for xylitol in comparison with no-gum control (Scheinin et al. 1985; Isokangas et al. 1988; Kandelman et al. 1988; Kandelman and Gagnon 1990; Mäkinen et al. 1995; Mäkinen et al. 1996; Alanen et al. 2000; Machiulskiene et al. 2001; Kovari et al. 2003) or placebo gum (Campus et al. 2013); PF varied from 22 to 114%. Eight of them analysed and reported the caries results for permanent teeth (Scheinin et al. 1985; Isokangas et al. 1988; Kandelman et al. 1988; Kandelman and Gagnon 1990; Mäkinen et al. 1995; Alanen et al. 2000; Machiulskiene et al. 2001; Campus et al. 2013); PF varied between 32 and 114%. These eight studies were carried out in high or moderate caries level subjects. One of the evaluated chewing gum studies reported the caries results in a combination for permanent and deciduous dentitions (Kovari et al. 2003). The study compared the use of xylitol chewing gum with toothbrushing in a kindergarten setting. A statistically significant (PF = 22%) but clinically minor difference was found in favour of the xylitol group. The study used the proportion of children with dmfs/DMFS > 0 as a measure of caries prevalence, and the baseline caries level was low. Of the ten evaluated chewing gum studies, the last (Mäkinen et al. 1996) compared xylitol stick and xylitol pellet chewing gums with no gum, and reported the results for deciduous dentition. The xylitol chewing gums reduced caries compared to no gum (PF = 47% and 63%). The baseline caries level of the study was high (Mäkinen et al. 1996).

In four chewing gum trials, xylitol gum was compared with a polyol gum. In three of them, the caries reduction was higher in the xylitol gum group compared to the control sorbitol/polyol gum group. In the two trials by Mäkinen et al. (1995, 1996), the first one showed significantly lower caries increments (permanent dentitions) for the two xylitol gums compared with the two sorbitol control gums. In the second trial (Mäkinen et al. 1996), xylitol and sorbitol gums reduced caries occurrence (primary dentitions) compared to no gum, but no differences between the xylitol and sorbitol gums were found. The study by Campus et al. (2013) showed a significantly lower caries increment for the xylitol gum group compared to the polyol mixture control group. Also, the results of the study by Machiulskiene et al. (2001) indicate that the caries-preventive effect of xylitol gum differed from the control sorbitol gum.

Six xylitol candy studies analysed a total of 1,023 subjects (Alanen et al. 2000; Oscarson et al. 2006; Stecksén-Blicks et al. 2008; Lenkkeri et al. 2012; Honkala et al. 2014; Lee et al. 2015). Only one of them (Alanen et al. 2000) reported a statistically significant preventive effect (61%) in comparison with no-candy control group. Within this study the xylitol candy (a chewable lozenge) and chewing gum groups did not differ in relation to caries increment. The study was carried out in a high-caries population and results were reported for permanent dentition (Alanen et al. 2000). Four of the remaining five xylitol candy studies did not report any statistically significant preventive effect in comparison with no-treatment control; PF varied from -18% to 33%. Three of them reported the caries results for permanent teeth (Stecksén-Blicks et al. 2008; Lenkkeri et al. 2012; Lee et al. 2015). One of them (Stecksén-Blicks et al. 2008) evaluated the effect of xylitol lozenges (relatively low daily dose of 2.5 g) on proximal caries in selected high-caries risk subjects. The study was considered to provide limited evidence due to the control group being formed of subjects who did not consent to participation in the intervention but agreed to participate in the examinations, as well as due to high risk of having an insensitive measure for caries increment. The second xylitol lozenge study (Lenkkeri et al. 2012) with no preventive effect was carried out in a very low-caries level population in an area of natural fluoridation. The third one (Lee et al. 2015) was carried out in very young children with low number of permanent teeth at risk, and very low-caries increments also in the control group, most probably due to intensive prevention given to all subjects. The study suffered from very high attrition of more than 50%. Lee et al. (2015) also reported results separately for deciduous teeth, with similar outcome. The fourth xylitol candy study with no significant preventive effect detected (Oscarson et al. 2006) had initially 2-year-old subjects in a low-caries level population, and the daily xylitol dosage had been very low, less than 1 g per day (Oscarson et al. 2006).

In addition, one xylitol candy study (Honkala et al. 2014) compared the relative preventive effect of xylitol and sorbitol candies and found no significant difference in dentin caries development between the xylitol and sorbitol groups. The study suffers from the lack of a no-candy control group. Caries was measured as the sum of dmfs + DMFS, which due to exfoliation of deciduous teeth is not a relevant measure in originally 7–9-year-old subjects during the 3-year follow-up period.

### Adverse effects

Possible adverse effects connected with the use of the test and control products were recorded and reported in seven of the 15 studies (Isokangas et al. 1988; Mäkinen et al. 1995; Alanen et al. 2000; Stecksén-Blicks et al. 2008; Lenkkeri et al. 2012; Campus et al. 2013; Lee et al. 2015). No side-effects were reported in three of these studies (Mäkinen et al. 1995; Alanen et al. 2000; Campus et al. 2013). In the study by Isokangas et al. (1988), four subjects in the xylitol group reported that they interrupted the study based on gastrointestinal problems. In the study by Stecksén-Blicks et al. (2008), no adverse effects were reported but two children interrupted the study during the first month due to insecurities associated with the intervention. Lenkkeri et al. (2012) reported that one child interrupted the study based on gastrointestinal discomfort. In the study by Lee et al. (2015), no adverse events or major side-effects were reported, but 17 children (no information on the group) experienced mild gastrointestinal discomfort and dropped out of the study.

## Discussion

The main finding of the present review is that adding xylitol chewing gum to the daily diet had a caries-reducing effect. The effect could not be demonstrated for xylitol candies. In the evaluated ten chewing gum studies conducted with xylitol gum or confectionery that included xylitol gum, xylitol consumption reduced caries occurrence in subjects with a high or moderate caries level at study baseline when compared with no treatment. In the permanent dentitions, the prevented fraction of caries increment varied greatly, but was consistently in favour of using xylitol gum. In nine trials the preventive fraction was more than 30%, which can be considered clinically significant (Scheinin et al. 1985; Isokangas et al. 1988; Kandelman et al. 1988; Kandelman and Gagnon 1990; Mäkinen et al. 1995; Mäkinen et al. 1996; Alanen et al. 2000; Machiulskiene et al. 2001; Campus et al. 2013). Four of the ten xylitol gum trials evaluated in the present review had an additional sorbitol or polyol gum control enabling the evaluation of specific xylitol effects. Three of these four trials suggested specific caries-reducing effects for the xylitol gum differing from those of the sorbitol/polyol gums used as controls.

Chewing xylitol gum not only stimulates the flow of saliva both based on a “chewing effect” but also the sweetness of xylitol. These effects contribute to the total or overall caries-preventive effects of xylitol chewing gums. Some studies have attributed the effects of xylitol gum on caries occurrence mainly to the chewing effect (Machiulskiene et al. 2001; Lingström et al. 2003). However, xylitol has reduced caries occurrence significantly also when administered as a syrup (Milgrom et al. 2009) and tooth wipes (Zhan et al. 2012) not involving chewing. Xylitol differs in some respects from polyols like sorbitol and maltitol used commonly in chewing gums. Xylitol is not fermented by oral microorganisms, whilst six-carbon polyols like sorbitol are slowly fermented by plaque bacteria (Havenaar et al. 1978). Thus, after xylitol gum chewing there is no drop in the pH of the dental plaque favouring remineralization. Earlier studies have also suggested that habitual consumption of xylitol in the form of chewing gum, reduces the counts of mutans streptococci and the amount of plaque. The reduction seems to differ from that found for sorbitol or polyol mixture gums (Söderling and Pienihäkkinen 2020; 2022). These results support the idea of specific caries-reducing effects of xylitol chewing gum.

The results of our review on xylitol chewing gums and candies are in line with many earlier systematic reviews (Deshpande and Jadad 2008; Rethman et al. 2011; Janakiram et al. 2017; Marghalani et al. 2017; ALHumaid and Bamashmous 2022). These reviews had a clinical and practical approach to the topic. They included comments like xylitol chewing gums and candies being used in self-care by the patients, and that using polyol-containing chewing gum should be a part of maintaining normal oral hygiene. The results of the present review, however, differ from the results of the earlier reviews by Lingström et al. (2003), Riley et al. (2015) and Mota et al. (2021). The review by Lingström et al. (2003) evaluated the effects of dietary changes in prevention of dental caries. The evaluation criteria were strict. The authors concluded that the evidence for the use of xylitol or sorbitol in chewing gum is inconclusive as studies with a high level of evidence were not available. The technically skilful Cochrane review on different xylitol-containing products for preventing dental caries included ten papers for evaluation (Riley et al. 2015). No chewing gum studies were evaluated. Furthermore, five of these the evaluated studies were conducted with very low daily xylitol doses and one in subjects with a very low-caries level (Riley et al. 2015). The review by Mota et al. (2021) evaluated five randomised clinical trials and compared prevention with xylitol chewing gum with prevention strategies or effective measures like sealants. Based on conflicting results, limitations, and inconsistencies of the evaluated studies the authors concluded that there is insufficient evidence to support the use of high-concentration xylitol gums for prevention of caries. These earlier published reviews emphasised inclusion of only randomised clinical trials, sought for a specific xylitol effect or excluded studies using a control group with no intervention. The different approaches may largely explain why the outcomes of these three reviews differed from those of our review. However, all the earlier and also the present review agree that more well designed clinical trials are needed on the topic.

For xylitol candies the results were not as uniform. A chewable lozenge was used as the xylitol vehicle in three studies. In the study by Alanen et al. (2000), its caries-reducing effect was similar to the xylitol chewing gum used in the trial. However, in two studies (Lenkkeri et al. 2012; Oscarson et al. 2006), both carried out in a low-caries level population, no effect on caries occurrence was found for the chewable lozenge. Low-daily doses of xylitol, 0.5–2.5 g, were reported in two studies (Stecksén-Blicks et al. 2008; Oscarson et al. 2006). With candies/lozenges, also the size and dissolvability of the product influence how xylitol is spread from the product to the saliva. Small lozenges may dissolve without xylitol accessing the plaque. In the study by Stecksén-Blicks et al. (2008), both the daily dose of xylitol and the size of the lozenge were small, and no effect was found on caries occurrence. Also, bigger products like compressed lozenges (Honkala et al. 2014) or gummy bears (Lee et al. 2015) may show rapid clearance from the oral cavity affecting the outcome of the trial. However, neither in the study of Honkala et al. (2014), without a no-product control group, nor in the trial of Lee et al. (2015), with an intensive prevention programme possibly masking the results, is it possible to evaluate how these xylitol products worked. Thus, gum appears to be effective as a xylitol vehicle compared to candies in reducing caries. Xylitol gums seem to be better than candies also in reducing the amount of plaque (Söderling and Pienihäkkinen 2022), for example. Chewing xylitol gum stimulates saliva secretion and spreads xylitol with saliva, to access dental plaque and teeth. Xylitol dissolves from a chewing gum with a high-concentration peak at 1 min, the bulk of xylitol being dissolved at 3-min gum chewing (Lif Holgerson et al. 2006). A rather short chewing time, approximately 5 min, has been suggested to optimise the effects on xylitol gum chewing on oral health (Söderling and Pienihäkkinen 2022).

Xylitol shows no retention to the oral cavity, which may explain why there appears to be a dose–response relationship in the beneficial effects of xylitol on oral health. Daily xylitol doses of 5–6 g or more with a consumption frequency of three times a day or more appear to be effective in reducing counts of mutans streptococci and the amount of plaque (Milgrom et al. 2006; Ly et al. 2006). Also, for caries reduction, the daily xylitol dose appears to be important. The dose–response relationship for xylitol was first suggested in the trial by Isokangas et al. (1988), included in the present review. In most chewing gum studies (Scheinin et al. 1985; Isokangas et al. 1988; Kandelman et al. 1988; Mäkinen et al. 1995; Mäkinen et al. 1996; Alanen et al. 2000; Campus et al. 2013), the daily xylitol doses could be considered high enough to achieve “xylitol effects”, and clinically significant prevented fractions were found in the evaluation. Only in one study with a daily dose of 2.5 g, only a small beneficial effect of xylitol gum chewing was detected (Kovari et al. 2003). In review articles on xylitol and caries, the authors may fail to acknowledge the importance of the xylitol dose as a confounding factor (Riley et al. 2015; Newton et al. 2020; He et al. 2023).

Digestive disorders are often connected in the literature with polyol consumption. Xylitol belongs to FODMAP (fermentable oligo-, di-, monosaccharides and polyols) substances which may not suit persons with a tendency for digestive disorders. For dental benefits relatively small daily doses of xylitol, 5–6 g/day, are recommended (Söderling 2009). In fact, complaints about digestive discomfort in xylitol studies are rare (Mäkinen 2016; Söderling and Pienihäkkinen 2020), even though in some older studies high daily xylitol doses of 14–20 g/day were consumed (Scheinin et al. 1985; Kandelman et al. 1988). Also, the results of our review support the idea of xylitol being well tolerated.

The caries levels of the subjects had a strong impact on the outcome of the evaluated xylitol trials. Nine of the ten chewing gum studies were carried out in subjects with high or moderate caries levels, and all of them showed a clinically significant caries-reducing effect for xylitol gum. The trial by Kovari et al. (2003) was the only xylitol gum study conducted in a low-caries population; however, a small but statistically significant caries reduction was found in the xylitol gum group. In high-caries populations, the proportion of subjects manifesting the disease is high. In addition, during the follow-up period the risk to develop new or cavitated lesions is high. The slopes of the control groups of four chewing gum studies (Scheinin et al. 1985; Isokangas et al. 1988; Mäkinen et al. 1995; Alanen et al. 2000) illustrate this well (Fig. 2): the studies have high baseline caries levels as well as high-caries increments. Clinically, this means that there is space for a beneficial change or an intervention to have effect. This in turn leads to high statistical power in the between-group comparisons. In fact, the above four studies showed clinically significant caries reductions in the xylitol gum groups. The caries level at study baseline is clearly of importance for the outcome of caries trials, as earlier discussed by Antonio et al. (2011). It is surprising that many reviews on effects of xylitol on caries occurrence neglect to acknowledge the caries level at baseline as a confounding factor (Mickenautsch et al. 2007, Deshpande and Jadad 2008; Riley et al. 2015; Janakiram et al. 2017; Marghalani et al. 2017; ALHumaid and Bamashmous 2022; He et al. 2023).

With decreasing caries level, the average number of decayed tooth surfaces, teeth and dentitions decrease, and the proportion of subjects with no disease and those with no new decay increases. In clinical trials with dental caries as an outcome measure this may cause difficulties: the DMF values are not normally distributed, the mean value does not describe the population well, statistical power decreases, and so forth. At low-caries level, the strong polarisation of the disease may also result in difficulties in measuring the disease: the crucial question is whether caries should be measured on a subject or on a tooth/surface level. The slopes of the control groups of two of the evaluated candy studies (Lenkkeri et al. 2012; Lee et al. 2015) were almost horizontal, leaving no space for the intervention effect (Fig. 2). In populations with very low-caries level, it seems difficult or impossible to show evidence for the effectiveness of a preventive measure. Nevertheless, the health-maintaining effect of the measure does exist. Interestingly, the trial by Lee et al. (2015) was conducted in a selected high-caries level population, but the extensive prevention programme given to both the xylitol and placebo candy groups apparently masked any effects of the xylitol intervention. If a study is carried out in subjects with a low baseline caries level and with a low daily dose of xylitol, it is no surprise that caries increments in the xylitol and control groups are low during the follow-up. A good example of such a study is the Oscarson et al. trial (2006).

Caries on smooth buccal and lingual surfaces of teeth has been considered most sensitive to preventive measures (Ruiken et al. 1986). Of the analysed xylitol studies, surface-specific analyses had been carried out in three studies (Scheinin et al. 1985; Isokangas et al. 1988; Kandelman et al. 1990). In the Canadian study with high-caries level, about half of the total caries burden was developed on buccal and lingual surfaces (Kandelman et al. 1990). The prevented fraction was reported to be highest on these surfaces and clearly less on proximal and occlusal surfaces. In Hungarian studies (high-caries level) (Scheinin et al. 1985) surface-related increment rates were clearly highest on the occlusal surfaces, and low on buccal and lingual surfaces and on proximal surfaces. The reduction in the caries rate related to the use of xylitol gum/confectionery was reported for only smooth surfaces, i.e. buccal and lingual, and proximal surfaces. In the study carried out in Finland (moderate caries level), significant reduction in caries increment (3-year) in the xylitol group was reported for all surface types, in selected high-risk subjects (Isokangas et al. 1988). However, it should be noted, that even in the control group, the majority of caries increment for buccal and lingual surfaces was pit and fissure lesions and minority on smooth surface (Isokangas et al. 1988). When all children were analysed together, corresponding significant reduction (2-year increment) was demonstrated only for occlusal surfaces. This was most probably due to a very low number of proximal lesions observed during the 2-year follow-up (Isokangas et al. 1988). In independent, long-term post-trial analyses (about 10 years), the evaluation of timing and number of restorations in these subjects was carried out using survival analysis methods (Virtanen et al. 1996). The results suggested that the first restorative treatment took place significantly later in the xylitol than in the control group. The preventive effect was most pronounced in teeth which erupted during the trial and for the proximal and other smooth surfaces, e.g. on proximal surfaces of upper incisors and second premolars (Virtanen et al. 1996). These observations showed the challenges in clinical trials with dental caries as an outcome measure: important benefits may only be detected in long-term follow-up. One example of such long-term benefit was related to maternal xylitol use, which was demonstrated to be associated with both reduced caries prevalence and the need for restorative treatment in the 10-year-old children of these mothers (Laitala et al. 2012). The surface-specific analyses suggest that xylitol gum use could work with the highest benefit in subjects with active incipient caries lesions detected on buccal or lingual surfaces of teeth.

The lack of high-quality original studies is the main limitation of the present systematic review. Nowadays, the study criteria in quality assessment emphasise randomisation and blinding. The evaluated studies were published within a broad time frame and ranked to be of relatively low quality (six fair-quality and nine low-quality studies). They were also, as expected, very heterogenous, making a meta-analysis or other statistical syntheses difficult or impossible to perform and interpret. However, interpretation of outcomes in relation to caries level of the subjects is a strength of this review. The original studies had some strengths, too. First of all, in many old studies, caries as a disease affected practically all subjects, and all surface types. In addition, in many trials, e.g. in the oldest field studies (Scheinin et al. 1985; Kandelman et al. 1988; Kandelman and Gagnon 1990; Mäkinen et al. 1995, 1996), pains were taken to ensure that the dentists participating in the caries registration were well calibrated—an important factor not taken into consideration in the present systematic reviews. Many old studies had also a relatively transparent way of reporting the results: on subject, on tooth and on surface level, which can be considered strengths of the studies. For instance, the above surface level evaluations (Scheinin et al. 1985; Isokangas et al. 1988; Kandelman et al. 1990) to some extent explain the arrestment of dental caries, and the general transparency in reporting increases the credibility of the outcomes.

Future studies on xylitol, polyols and other preventive measures should thus include selection of high-risk subjects and preferably the use of some specific diagnostic tools (e.g. utilising laser fluorescence) to enable short-term clinical studies and a better control of confounders. The progression or arrestment of active caries lesions could then be much more closely associated with different preventive measures than can be achieved in clinical trials covering several years. Inclusion of a placebo control is essential when studying, e.g. specific effects of polyols, but in the evaluation of their clinical significance, the study setting should include also the no-intervention arm. We consider that first it is important to show whether the preventive effect exists or not. For any preventive measure (also for xylitol) the effect can be detected when the subjects at study baseline have at least a moderate risk for developing caries. Second, the practical/clinical value of the preventive measure should be evaluated based, e.g. on the type of the preventive measure, the magnitude of the preventive effect, the mode of action and on the associated costs and adverse effects. The practical value strongly depends on the caries level of the population and on the patient’s individual risk for caries. The results obtained in subjects with high/moderate caries level are necessarily not generalizable to low-caries level populations. However, the results can and should be utilised in self-care for the maintenance of oral health and in controlling dental caries in subjects with high or moderate risk for caries. National and regional guidelines may help dental professionals in their decision making. Clinical recommendations for professionals and for lay people should be based on systematic reviews with clinically relevant research questions, combined with knowledge and understanding of the disease, as well as that of the mechanism of action of the preventive measures.

## Conclusion

The findings of the present review suggest that a clinically significant caries-reducing effect of adding xylitol chewing gum to the daily diet has been well demonstrated in children and adolescents with a high or moderate caries level at baseline. The results also suggest that the caries-reducing effects of xylitol gum may differ from sorbitol/polyol gums, but more research is needed on this topic.

A caries-reducing effect could not be demonstrated for xylitol candies, but more research is needed also on this topic, especially in high-caries risk subjects.

This knowledge may provide dental professionals with confidence in their recommendations of xylitol use to their high-caries risk patients in particular. The surface-specific analyses suggest that xylitol gum use could work with the highest benefit in subjects with active incipient caries lesions detected on buccal or lingual surfaces of teeth. Xylitol use is, anyhow, one piece in a puzzle of caries prevention to be used together with tooth brushing with fluoride toothpaste twice a day and restriction of sucrose intake.
